# Pregnancy With SLE and Fetal Congenital Heart Block: A Case Report

**DOI:** 10.4021/cr278w

**Published:** 2013-07-11

**Authors:** Suman Puri, Puneet Pooni, Bishav Mohan, Vidushi Bindal, Sugam Verma, Sumati Verma, Rajiv Kumar Gupta

**Affiliations:** a Department of Obstetrics and Gynaecology, Dayanand Medical College and Hospital, Ludhiana, India; b Department of Pediatrics, Dayanand Medical College and Hospital, Ludhiana, India; c Department of Cardiology, Hero DMC Heart Centre - Dayanand Medical College and Hospital, Ludhiana, India; d Dayanand Medical College and Hospital, Ludhiana, India; e Department of Cardiovascular and Thoracic Surgery, Hero DMC Heart Centre - Dayanand Medical College and Hospital, Ludhiana, India

**Keywords:** SLE with pregnancy, Fetal congenital heart block, Fetal bradycardia

## Abstract

Autoimmune AV block is usually seen in association with autoimmune antibodies in mother that cross the placenta and damage the AV node of fetus. A 24-year-old primigravida, diagnosed to have SLE, at 25 weeks period of gestation found to have fetal bradycardia. Her ANA was moderately positive, SS-A (Ro) antibodies and SS-B (La) antibodies were positive. Fetal ECHO showed no structural defect but heart rate was 55 - 60 beats per minute. She was put on dexamethasone (4 mg/day). She was lost on follow up and presented at term in emergency with labor pains and fetal bradycardia, underwent a lower segment caesarean section. Baby underwent a temporary cardiac pacing within 10 hours of birth followed by permanent pacing on day 3 of birth. Baby is doing well on follow up. Neonates with isolated congenital heart block who are monitored antenatally and delivered in a planned fashion at an institution capable of early pacing can have favorable outcomes.

## Introduction

Congenital heart block can occur in fetal life due to maternal disease or due to congenital heart defect in fetus which can manifest at any time before or after birth. Congenital heart block in a structurally normal heart is isolated congenital heart block or with congenital heart disease is complex congenital heart block. Autoimmune AV block occurs in approximately 1 per 14,000 - 20,000 live births [1]. Isolated congenital heart blocks is usually seen in association with autoimmune antibodies in mother that cross the placenta and damage the AV node of the fetus and thus diffuse myocarditis and fibrosis. The mother can be completely asymptomatic in presence of these autoimmune antibodies or may have a diagnosis of an autoimmune disorder. Systemic lupus erythematosus (SLE) is a chronic inflammatory disease with multisystem involvement in which the tissues are damaged by autoantibodies and immune complexes. In 90% of cases the disease affects women, the incidence of SLE during the child bearing age being 1 in 500. The fetal effects are mainly prematurity, intrauterine growth restriction, neonatal lupus, and in extreme cases stillbirth. The most serious complication in the neonate is complete heart block, which occurs in approximately 2 percent of such pregnancies. Bradycardia is an indication for pacing in isolated congenital heart block. Patients without pacemaker may develop AV valve regurgitation, atrial rhythm disorders, thromboembolism, congestive heart failure and sudden death [2].

## Case Report

In 2012, a 24-year-old primigravida referred as a case of 25 weeks pregnancy with fetal bradycardia to Obstetrics and Gynecology OPD of a tertiary care centre in North India. She had a history of photosensitivity and oral ulcers off and on. On examination, she was thinly built, mildly anemic without any pedal edema, and normotensive with normal vital parameters. On abdominal examination, the uterus was about 24 weeks size. The fetal heart sound was located by CTG machine and rate being alarming, 68 beats/minute. Fetal movements were felt during examination. Her hemoglobin at admission was 9.8 g/dL and peripheral blood smear showed microcytic hypochromic picture with mild anisocytosis. Her blood sugar levels, kidney and liver function tests were within normal limits. She was VDRL negative. Her ESR was 60 mm, CRP 2.2 and TSH 3.05, DCT was negative. Immunological tests revealed serum ANA moderately positive, and SS-A (Ro) antibodies and SS-B (La) antibodies strongly positive and APTT 27.4 seconds (control 27 seconds). Ultrasound examination on admission, showed a single live fetus of 24 weeks maturity, adequate liquor, and fetal heart rate of 56 beats/minutes. Fetal echocardiography showed no structural defect but the fetal heart rate was 55 - 60 beats/minute suggestive of congenital heart block. Maternal echocardiography findings were within normal limits. Her history, clinical findings and investigations were suggestive of SLE as in accordance to the American Rheumatism Association she had 4 of 11 criteria present she was diagnosed as a case of SLE with pregnancy with congenital heart block in fetus. She and her husband were counseled regarding the prognosis of the fetus. She was put on medication with low dose aspirin (75 mg once a day), dexamethasone (4 mg/day), and iron, calcium and vitamin supplementation. She was lost on follow up. She reported back in emergency at 37 weeks with labor pains and fetal bradycardia due to congenital heart block. A lower segment cesarean section was performed on Dec 7, 2012 and a live female baby weighing 2.1 kg was born. The heart rate at birth was 60 beats/minute and APGAR score at 1 minute and 5 minutes was 6 and 9 respectively. Respiratory rate was 66/minute and a grade 3 systolic murmur was heard in left parasternal area. The baby was immediately transferred to the neonatal intensive care unit (NICU). ECG was done which showed signs of complete heart block. Cardiac consultation was taken and advised to monitor vitals. After 9 hours of birth, heart rate dropped to 48/minute. Hence, cardiac pacing was done in the CTVS unit of the same centre. Initially temporary pacing was done through trans-femoral route at about 10 hours of birth. Post procedure heart rate was 130/minute and baby was monitored in NICU. Permanent pacing was planned on Dec 10, 2012 (namely after 3 days of birth), done by sub-xiphoid approach. Pacemaker was put through right ventricle, connected to power source in the abdomen through subcutaneous plane. Post-operatively, baby required inotropic support (Dopamine and Dobutamine) and received one packed cell transfusion. Gradually as the general condition stabilized inotropes were tapered off and chest tube removed. Thus with the help of a multidisciplinary team, the patient was discharged after 2 weeks with a healthy baby accepting feeds and gaining weight.

## Discussion

Isolated congenital heart block is presumed to be caused by injury from the placental passage of maternal anti-Ro and anti-La (or related) antibodies, which are present in more than 90% of mothers during pregnancy or at the time of delivery. These autoantibodies damage the AV conduction tissue possibly by inflammation or direct ion channel interaction in the early stage and later by fibrosis [3-5]. The prognosis of isolated congenital heart block in patients presenting as fetuses or at birth is associated with higher morbidity and mortality rates (which may be as high as 30-50%) [6]. Risk factors for death in these patients are very low heart rate, low birth weight, premature gestation, hydrops fetalis, endocardial fibroelastosis, diminished ventricular function [1]. Patients who are diagnosed and treated in the neonatal period have a survival rate of 94% [1].

SLE with pregnancy is rare, the incidence being 1 in 1,600.The disease remains stable in about 30% but pregnancy at times may cause flare ups [7]. Identification of ANA is the best screening test. Anti-ds DNA is highly specific for SLE. Mothers with autoimmune antibodies should undergo regular fetal ultrasonographic assessments including fetal echocardiography as early as 16 weeks of gestation which can reveal varying degrees of AV block and further fetal monitoring should be performed to look for bradycardia, fetal distress, hydrops fetalis [1].

Regular and close monitoring for heart block and transplacental therapy with fluorinated steroids (dexamethasone) have shown satisfactory results as first evidence of heart block. Prophylactic therapy is not currently indicated as they may have maternal and fetal side effects [8-10]. Information on prenatal progression of the cardiac anomaly is important to plan perinatal management, as early pacemaker insertion may be required in some newborns. Temporary pacing can be achieved transcutaneously, transesophageally, or transvenously. Permanent pacemaker placement is eventually needed in most children with congenital heart block. The medical care of congenital heart block is currently focused on identifying the optimal timing of pacemaker therapy to ensure a positive outcome. Neonates with isolated congenital heart block who are monitored antenatally and delivered in a planned fashion at an institution capable of early pacing can have favourable outcomes. Planned early pacing of high risk neonate with isolated congenital heart block potentially reduces the consequences of bradycardia and asystole [11].

There is a 14% mortality rate before 3 months of age, the cumulative probability of survival at 3 year age is 79% and 635 of live born eventually require pacemakers [12].

### Conclusion

Management of fetus with congenital heart block is early diagnosis; close fetal monitoring, use of maternal steroids followed by planned delivery at an institution and early but staged pacing procedure.
